# Post-resuscitation diastolic blood pressure is a prognostic factor for outcomes of cardiac arrest patients: a multicenter retrospective registry-based analysis

**DOI:** 10.1186/s40560-022-00631-6

**Published:** 2022-08-06

**Authors:** Chien-Yu Chi, Min-Shan Tsai, Li-Kuo Kuo, Hsin-Hui Hsu, Wei-Chun Huang, Chih-Hung Lai, Herman Chih-Heng Chang, Chu-Lin Tsai, Chien-Hua Huang

**Affiliations:** 1 Department of Emergency Medicine, National Taiwan University Hospital Yunlin Branch, Yunlin, Taiwan; 2 Graduate Institute of Clinical Medicine, Medical College, National Taiwan University, Taipei, Taiwan; 3 Department of Emergency Medicine, National Taiwan University Hospital, #7, Chung-Shan South Road, Taipei, 100 Taiwan; 4 Department of Critical Care Medicine, MacKay Memorial Hospital, Taipei branch, Taiwan; 5 Department of Critical Care Medicine, Changhua Christian Hospital, Changhua, Taiwan; 6 Department of Critical Care Medicine, Kaohsiung Veterans General Hospital, Kaohsiung, Taiwan; 7 Cardiovascular Center, Taichung Veterans General Hospital, Taichung, Taiwan; 8 Department of Emergency and Critical Care Medicine, Fu-Jen Catholic University Hospital, New Taipei City, Taiwan

**Keywords:** Cardiac arrest, Post-cardiac arrest care, Diastolic blood pressure, Outcomes

## Abstract

**Background:**

Post-resuscitation hemodynamic level is associated with outcomes. This study was conducted to investigate if post-resuscitation diastolic blood pressure (DBP) is a favorable prognostic factor.

**Methods:**

Using TaIwan Network of Targeted Temperature ManagEment for CARDiac Arrest (TIMECARD) registry, we recruited adult patients who received targeted temperature management in nine medical centers between January 2014 and September 2019. After excluding patients with extracorporeal circulation support, 448 patients were analyzed. The first measured, single-point blood pressure after resuscitation was used for analysis. Study endpoints were survival to discharge and discharge with favorable neurologic outcomes (CPC 1–2). Multivariate analysis, area under the receiver operating characteristic curve (AUC), and generalized additive model (GAM) were used for analysis.

**Results:**

Among the 448 patients, 182 (40.7%) patients survived, and 89 (19.9%) patients had CPC 1–2. In the multivariate analysis, DBP > 70 mmHg was an independent factor for survival (adjusted odds ratio [aOR] 2.16, 95% confidence interval [CI, 1.41–3.31]) and > 80 mmHg was an independent factor for CPC 1–2 (aOR 2.04, 95% CI [1.14–3.66]). GAM confirmed that DBP > 80 mmHg was associated with a higher likelihood of CPC 1–2. In the exploratory analysis, patients with DBP > 80 mmHg had a significantly higher prevalence of cardiogenic cardiac arrest (*p* = 0.015) and initial shockable rhythm (*p* = 0.045).

**Conclusion:**

We found that DBP after resuscitation can predict outcomes, as a higher DBP level correlated with cardiogenic cardiac arrest.

## Background

Post-cardiac arrest care is crucial for arrested patients, especially for those not returning to baseline consciousness. One of the approaches is to optimize these patients’ hemodynamic status. Several observational studies have shown that hypotension in post-resuscitated patients is related to poor outcomes [1–6]. Current guidelines recommend maintaining mean blood pressure (MBP) higher than 65 mmHg or systolic blood pressure (SBP) higher than 90 mmHg [7, 8]. European Resuscitation Council guidelines also recommend an individualized hemodynamic target in the post-cardiac arrest care period [8].

The lower limit of cerebral autoregulation might become higher in post-resuscitated patients [9]. Targeting higher blood pressure (BP) might theoretically improve cerebral blood perfusion and neurologic outcomes [10]. However, current evidence is not conclusive, and several observational studies have reported controversial results [6, 11–17]. Recent randomized studies reported no difference in long-term cognitive function, serum neuron-specific enolase level, or brain image findings when targeting higher MBP levels during early post-cardiac arrest care [18–20]. However, these studies were conducted with small sample sizes.

Targeted treatment for diastolic blood pressure (DBP) level during the post-resuscitated period is less studied. The physiological significance of DBP differs from that of SBP. A higher DBP level indicates higher peripheral vascular resistance, better coronary vessel perfusion, and better survival in cardiogenic shock patients [21]. Only one study reported a correlation between DBP level during the first 6 h of care in an intensive care unit (ICU) and neurologic outcomes of cardiac arrest patients [22]. Recent studies have shown the prognostic value of DBP during cardiopulmonary resuscitation (CPR) in predicting successful resuscitation [23–25].

In this study, we aimed to demonstrate the correlation between patients’ outcomes and hemodynamic parameters when return of spontaneous circulation (ROSC) occurs. We hypothesized that DBP during ROSC is a prognostic factor for outcomes of cardiac arrest patients. We also sought to explain the possible pathophysiological relation between DBP and the outcomes.

## Methods

### Study population and setting

In January 2014, the Taiwan Society of Emergency and Critical Care Medicine launched the TaIwan Network of Targeted Temperature ManagEment for CARDiac Arrest (TIMECARD) registry to establish a study cohort of patients treated with targeted temperature management (TTM). The TIMECARD registry is a nationwide, multicenter, post-cardiac arrest care registry collecting information on patients with TTM in Taiwan. Enrolled patients included non-traumatic out-of-hospital cardiac arrest (OHCA) or in-hospital cardiac arrest (IHCA) adult survivors who were treated with TTM in nine medical centers in Taiwan [26].

Patients were resuscitated and treated according to current guidelines [7, 8]. Patients with sustained ROSC and remaining in a comatose state received TTM for less than 12 h in the ICU. Comatose state was defined by a Glasgow Coma Scale score of less than 8 or inability to obey commands. Cooling was performed using cooling blankets or venous cooling catheters according to the protocols in each hospital. Core body temperature was recorded using esophageal probes or venous catheters. The targeted temperature of 33 °C was achieved as soon as possible, maintained for 24 h, and followed by a slow rewarming stage until the body temperature reached 36.5 °C at a rate of 0.25 °C per hour. The hemodynamic status was recorded by non-invasive blood pressure (NIBP) monitoring in the emergency department (ED) or general ward and using an arterial catheter in the ICU.

Patients’ demographic factors, medical histories, and cardiac arrest variables were recorded in the registry according to the Utstein style after reviewing pre-hospital and hospital medical records [27]. In addition to Utstein style parameters, details of resuscitation, hemodynamic status during ROSC, new-onset complications during post-cardiac arrest care, performing of percutaneous coronary intervention (PCI), and extracorporeal membrane oxygenation (ECMO) during resuscitation were also recorded.

### Patient selection, data acquisition, and outcome measurements

In this study, the study population was retrospectively retrieved from the TIMECARD registry for the period of January 2014 to September 2019. Adult non-traumatic OHCA or IHCA survivors treated with TTM were included. Patients with a pre-arrest cerebral performance category (CPC) 3–4 were excluded from the study due to the neurologic endpoints of this study. Patients who received ECMO support during resuscitation were also excluded because hemodynamic monitoring was often influenced by extracorporeal pumping.

Patients’ demographic data, underlying comorbidities, cardiac arrest etiologies, initial rhythms, total CPR durations, total epinephrine dosages during CPR and performing of PCI were analyzed. The first measured, single-point BP after achieving ROSC was used for analysis. The BP was mostly monitored by NIBP because almost all cardiac arrests occurred in the ED or ward. SBP and DBP were recoded accordingly. MBP was estimated by the following formula: MBP = DBP + 1/3 (SBP – DBP). New-onset complications during post-cardiac arrest care, such as bleeding, severe infection, arrhythmia, seizure, and hypoglycemia were analyzed to explain the possible pathophysiology mechanism. If the abovementioned complications occurred within the first 7 days of ICU admission, then these complications were defined as new-onset complications. New-onset bleeding was defined as any visible bleeding with a need for further blood transfusion. New-onset severe infection was defined as the development of any new pneumonia patches, septic shock, or bacteremia. The endpoints of this study were survival to discharge and discharge with favorable neurologic outcomes (CPC 1–2).

### Statistical analysis

Continuous variables were converted to categorial subgroups using appropriate methods due to the non-normal distribution. Categorical variables were presented as case numbers and percentages. The Chi-square test or Fisher exact test was used for univariate analysis. Those variables with *p*-values < 0.1 were included in a stepwise logistic regression for predicting independent variables of outcomes. Independent variables were presented with adjusted odds ratios (aORs) and 95% confidence intervals (CIs). Receiver operating characteristic (ROC) curves for survival and favorable neurologic outcomes were plotted according to SBP, MBP, and DBP values. The generalized additive model (GAM) was used to visualize the association between DBP and favorable outcomes. The threshold DBP value for better outcomes was determined by testing incremental cut-off values of DBP until there was no difference between the two groups in the multivariate analysis. Finally, the threshold was used for stratifying the study population into two groups with high and low DBP values. Outcomes, characteristics, and post-cardiac arrest complications of these two groups were compared by exploratory analysis. All statistical analyses were performed using STATA software version 16 (StataCorp, TX, USA).

## Results

### Overview

**Fig. 1:**
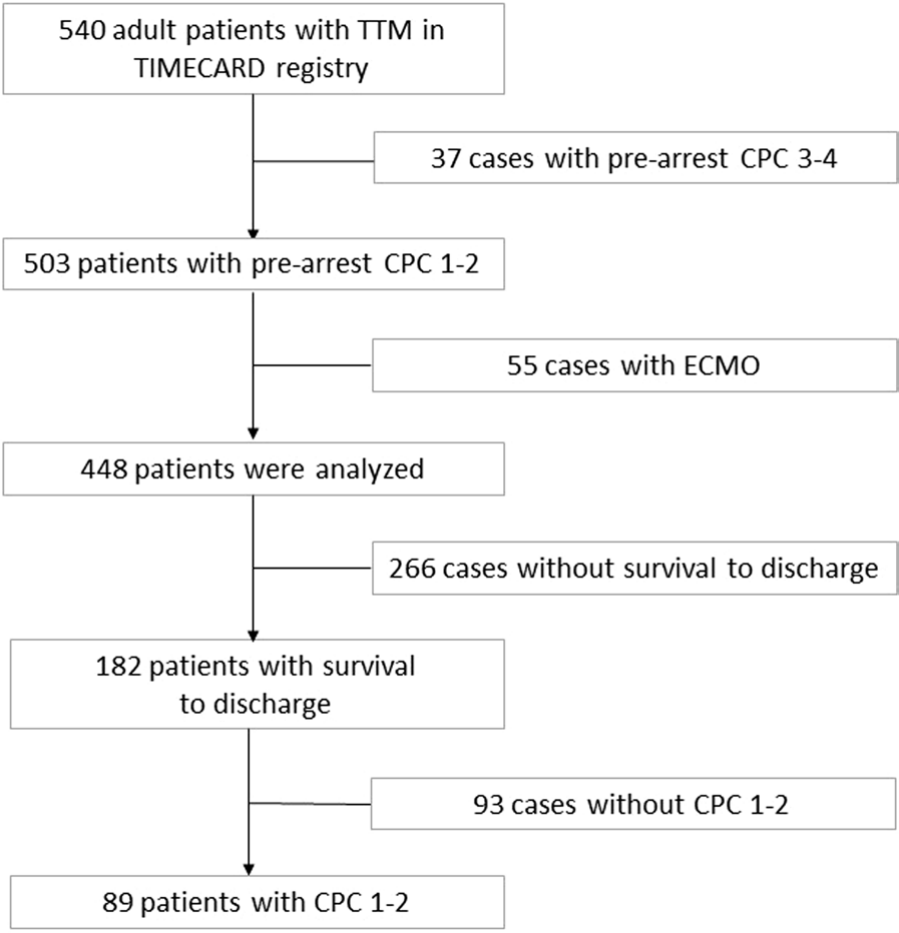
Study flow diagram. *TTM* targeted temperature management, *CPC* cerebral performance scale, *ECMO* extracorporeal membrane oxygenation

During the study period, a total of 540 patients were enrolled in the TIMECARD registry. Thirty-seven (7%) patients were excluded for pre-arrest CPC 3–4, and 55 patients (11.0%) were excluded for receiving ECMO. Thus, a total of 448 patients were included in the final analysis (Fig. 1). A total of 182 patients (40.6%) had survival to discharge, and 89 patients (19.9%) were discharged with favorable neurologic outcomes.

### Characteristics and predictive factors of survival to discharge

**Fig. 2:**
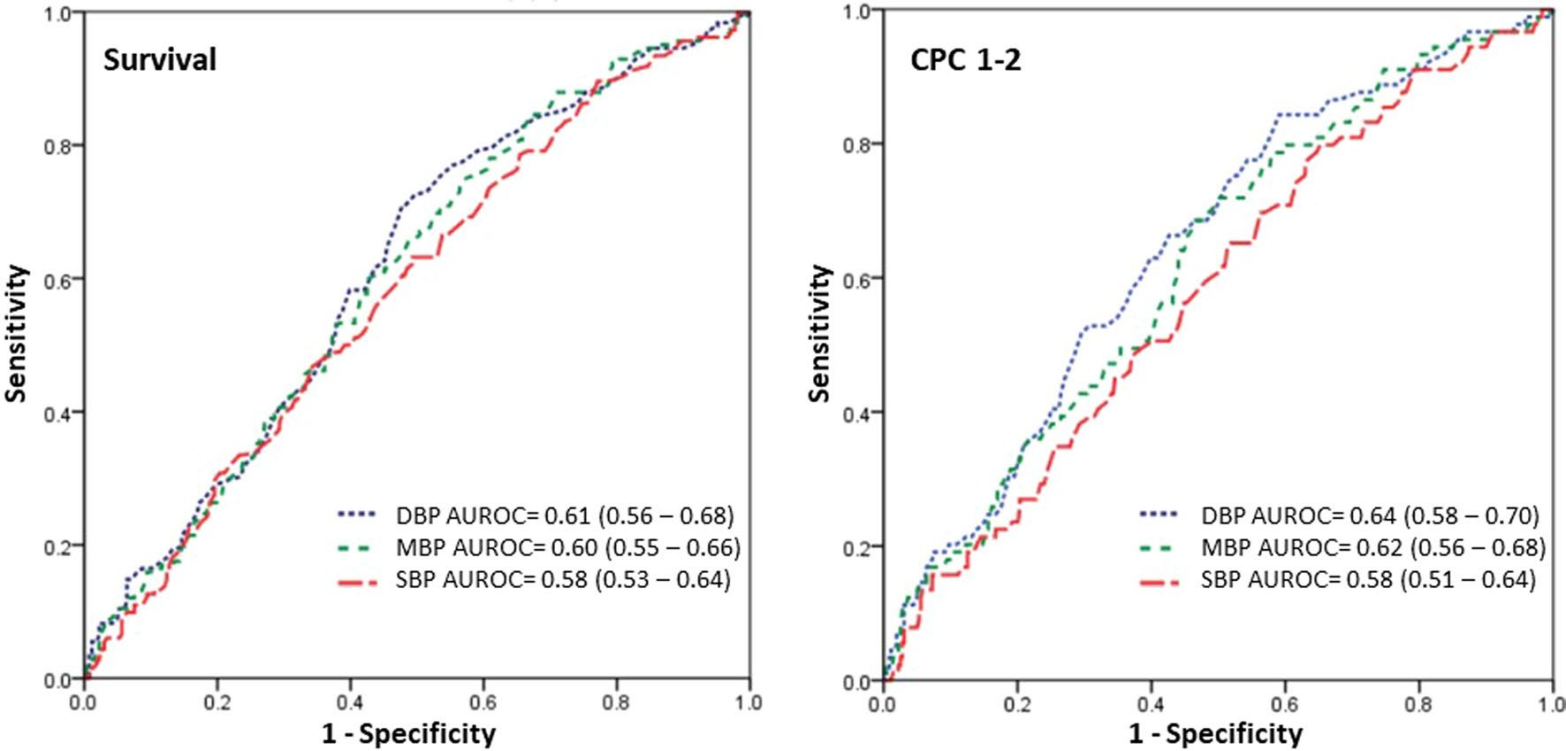
Receiver operating characteristic (ROC) curves of systolic blood pressure (SBP), mean blood pressure (MBP), and diastolic blood pressure (DBP) for outcomes. *AUROC* area under the receiver operating characteristic curve, *SBP* systolic blood pressure, *MBP* mean blood pressure, *DBP* diastolic blood pressure, *CPC* cerebral performance scale

The results of the univariate and multivariate analyzes for predictive factors of survival are listed in Table 1. In the univariate analysis, the ROSC DBP distributions of the survival and non-survival groups differed significantly (*p* < 0.001), while ROSC SBP showed no difference (*p* = 0.061). ROSC DBP had an area under the ROC (AUROC) of 0.61 (0.56–0.68) for survival to discharge, while ROSC SBP had an AUROC of 0.58 (0.53–0.64, Fig. 2). In the multivariate analysis, heart failure, end-stage renal disease, initial rhythm, epinephrine dosage, ROSC DBP, and PCI were independent factors for predicting survival to discharge. Patients with ROSC DBP in the range of 70–80 mmHg [aOR: 3.31 (1.65–6.64)], 80–90 mmHg [aOR: 2.13 (1.03–4.40)], and > 90 mmHg [aOR: 1.95 (1.11–3.42)] showed a greater chance of survival than patients with ROSC DBP < 60 mmHg.

**Table 1:** Univariate and multivariate analyses for predictive factors of survival

	Survival to discharge (*n*,%)	Survival to discharge (*n*,%)	*p*-value	Adjusted OR	*p*-value
	Yes, *n* = 182	No, *n* = 266		(95% CI)	
Age (years)			0.004*		
< 40	21 (11.5%)	14 (5.3%)			
40–60	60 (33.0%)	62 (23.2%)			
60–80	72 (39.6%)	132 (49.6%)			
> 80	29 (15.9%)	58 (21.8%)			
Origin			0.597		
OHCA	152 (83.5%)	217 (81.6%)			
IHCA	30 (16.5%)	49 (18.4%)			
Gender, male	123 (67.6%)	163 (61.3%)	0.173		
DM	60 (33.0%)	123 (46.2%)	0.005*		
HTN	95 (52.2%)	152 (57.1%)	0.301		
CAD	45 (24.7%)	76 (28.6%)	0.368		
HF	23 (12.6%)	68 (25.6%)	0.001*	0.51 (0.29–0.95)*	0.021*
COPD	21 (11.5%)	31 (11.7%)	0.97		
ESRD	12 (6.6%)	46 (17.3%)	0.001*	0.43 (0.21–0.89)*	0.022*
Malignancy	21 (11.5%)	38 (14.3%)	0.398		
Witness	154 (84.6%)	198 (74.4%)	0.010*		
Bystander CPR	132 (72.5%)	161 (60.5%)	0.009*		
Cardiac etiology	108 (59.3%)	109 (41.0%)	< 0.001*		
Initial rhythm			< 0.001*		0.001*
Asystole	46 (25.3%)	141 (53.0%)		Reference	
PEA	51 (28.0%)	64 (24.4%)		2.23 (1.32–3.77)*	
VT/VF	85 (46.7%)	60 (22.6%)		2.69 (1.54–4.69)*	
CPR duration (min)			0.105		
< 10	46 (25.3%)	54 (20.3%)			
Oct-20	57 (31.3%)	70 (26.3%)			
20–30	41 (22.5%)	60 (22.6%)			
> 30	38 (20.9%)	82 (30.8%)			
Epinephrine dosage (mg)			0.001*		0.038*
0–2	114 (62.6%)	121 (45.5%)		Reference	
2–4	41 (22.5%)	68 (25.6%)		0.86 (0.51–1.44)	
4–6	12 (6.6%)	27 (10.2%)		0.70 (0.31–1.55)	
> 6	15 (8.2%)	50 (18.8%)		0.36 (0.18–0.72)*	
ROSC SBP (mmHg)			0.061		
< 100	32 (17.6%)	77 (28.9%)			
100–110	13 (7.1%)	21 (7.9%)			
110–120	16 (8.8%)	23 (8.6%)			
120–130	13 (7.1%)	20 (7.5%)			
> 130	108 (59.3%)	125 (47.0%)			
ROSC DBP (mmHg)			< 0.001*		0.005*
< 60	37 (20.3%)	103 (38.7%)		Reference	
60–70	27 (14.8%)	42 (15.8%)		1.14 (0.58–2.24)	
70–80	32 (17.6%)	27 (10.2%)		3.31 (1.65–6.64)*	
80–90	26 (14.3%)	28 (10.5%)		2.13 (1.03–4.40)*	
> 90	60 (33.0%)	66 (24.8%)		1.95 (1.11–3.42)*	
ROSC MBP (mmHg)			0.003*		
< 80	43 (23.6%)	105 (39.5%)			
80–90	19 (10.4%)	31 (11.7%)			
90–100	35 (19.2%)	34 (12.8%)			
100–110	14 (7.7%)	24 (9.0%)			
> 110	71 (39.0%)	72 (27.1%)			
PCI	78 (42.9%)	45 (16.9%)	< 0.001*	2.62 (1.54–4.47)	< 0.001*

*OHCA* out-of-hospital cardiac arrest, *IHCA* in-hospital cardiac arrest, *DM* diabetes mellitus, *HTN* hypertension, *CAD* coronary artery disease, *HF* heart failure, *COPD* chronic obstructive pulmonary disease, *ESRD* end-stage renal disease, *CPR* cardiopulmonary resuscitation, *PEA* pulseless electrical activity, *VT* ventricular arrhythmia, *VF* ventricular fibrillation, *ROSC* return of spontaneous circulation, *SBP* systolic blood pressure, *DBP* diastolic blood pressure, *MBP* mean blood pressure, *PCI* percutaneous coronary intervention

^*^Indicates *p*-value < 0.05

### Characteristics and predictive factors of favorable neurologic outcomes

The results of the univariate and multivariate analyses for predictive factors of favorable neurologic outcomes when discharged are listed in Table 2. In the univariate analysis, the ROSC DBP distributions of the groups with favorable and non-favorable neurologic outcomes differed significantly (*p* = 0.003), while ROSC SBP showed no difference (*p* = 0.116). ROSC DBP had an AUROC of 0.64 (0.58–0.70) for CPC 1–2, while ROSC SBP had an AUROC of 0.58 (0.51–0.64, Fig. 2). In the multivariate analysis, diabetes mellitus, malignancy, patients’ bystander status, initial rhythm, CPR duration, and epinephrine dosage were independent factors for predicting favorable neurologic outcomes. Patients with ROSC DBP > 90 mmHg [aOR: 2.73 (1.22–6.10)] showed a greater chance of favorable neurologic outcomes than patients with ROSC DBP < 60 mmHg.

**Table 2:** Univariate and multivariate analyses for predictive factors of CPC 1–2

	CPC 1–2 (*n*,%)	CPC 1–2 (*n*,%)	*p*-value	Adjusted OR	*p*-value
	Yes, *n* = 89	No, *n* = 359		(95% CI)	
Age (years)			< 0.001*		
< 40	16 (18.0%)	19 (5.3%)			
40–60	30 (33.7%)	92 (25.6%)			
60–80	31 (34.8%)	173 (48.2%)			
> 80	12 (13.5%)	75 (20.9%)			
Origin			0.924		
OHCA	73 (82.0%)	296 (82.5%)			
IHCA	16 (18.0%)	63 (17.5%)			
Gender, male	63 (70.8%)	223 (62.1%)	0.128		
DM	25 (28.1%)	158 (44.0%)	0.006*	0.53 (0.28–0.98)	0.044*
HTN	48 (53.9%)	199 (55.4%)	0.799		
CAD	26 (29.2%)	95 (26.5%)	0.601		
HF	12 (13.5%)	79 (22.0%)	0.074		
COPD	4 (4.5%)	48 (13.4%)	0.019*		
ESRD	7 (7.9%)	51 (14.2%)	0.157		
Malignancy	6 (6.7%)	53 (14.8%)	0.045*	0.26 (0.09–0.71)	0.009*
Witness	80 (89.9%)	272 (75.8%)	0.004*		
Bystander CPR	71 (79.8%)	222 (61.8%)	0.001*	2.88 (1.41–5.87)	0.004*
Cardiac etiology	64 (71.9%)	153 (42.6%)	< 0.001*		
Initial rhythm			< 0.001*		< 0.001*
Asystole	6 (6.7%)	181 (50.4%)		Reference	
PEA	25 (28.1%)	91 (25.3%)		8.59 (3.18–23.24)*	
VT/VF	58 (65.2%)	87 (24.2%)		17.78 (6.45–48.95)*	
CPR duration (min)			0.006*		0.008*
< 10	30 (33.7%)	70 (19.5%)		Reference	
Oct-20	28 (31.5%)	99 (27.6%)		0.46 (0.21–1.02)	
20–30	17 (19.1%)	84 (23.4%)		0.28 (0.12–0.67)*	
> 30	14 (15.7%)	106 (29.5%)		0.24 (0.10–0.62)*	
Epinephrine dosage (mg)			< 0.001*		0.015*
0–2	70 (78.7%)	165 (46.0%)		Reference	
2–4	11 (12.4%)	98 (27.3%)		0.33 (0.15–0.75)*	
4–6	3 (3.4%)	36 (10.0%)		0.39 (0.10–1.50)	
> 6	5 (5.6%)	60 (16.7%)		0.34 (0.11–1.07)	
ROSC SBP (mmHg)			0.116		
< 100	15 (16.9%)	94 (26.2%)			
100–110	3 (3.4%)	31 (8.6%)			
110–120	9 (10.1%)	30 (8.4%)			
120–130	8 (9.0%)	25 (7.0%)			
> 130	54 (60.7%)	179 (49.9%)			
ROSC DBP (mmHg)			0.003*		0.091
< 60	14 (15.7%)	126 (35.1%)		Reference	
60–70	13 (14.6%)	56 (15.6%)		1.17 (0.44–3.12)	
70–80	12 (13.5%)	47 (13.1%)		2.33 (0.84–6.47)	
80–90	14 (15.7%)	40 (11.1%)		2.20 (0.79–6.14)	
> 90	36 (40.4%)	90 (25.1%)		2.73 (1.22–6.10)*	
ROSC MBP (mmHg)			0.006*		
< 80	18 (20.2%)	130 (36.2%)			
80–90	7 (7.9%)	43 (12.0%)			
90–100	20 (22.5%)	49 (13.6%)			
100–110	6 (6.7%)	32 (8.9%)			
> 110	38 (42.7%)	105 (29.2%)			
PCI	51 (57.3%)	72 (20.1%)	< 0.001*		

*CPC* cerebral performance category, *OHCA* out-of-hospital cardiac arrest, *IHCA* in-hospital cardiac arrest, *DM *diabetes mellitus, *HTN* hypertension, *CAD* coronary artery disease, *HF* heart failure, *COPD* chronic obstructive pulmonary disease, *ESRD* end-stage renal disease, *CPR* cardiopulmonary resuscitation, *PEA* pulseless electrical activity, *VT* ventricular arrhythmia, *VF* ventricular fibrillation, *ROSC* return of spontaneous circulation, *SBP* systolic blood pressure, *DBP* diastolic blood pressure, *MBP* mean blood pressure, *PCI* percutaneous coronary intervention

^*^Indicates *p*-value < 0.05

### Testing of DBP thresholds for favorable outcomes

**Fig. 3:**
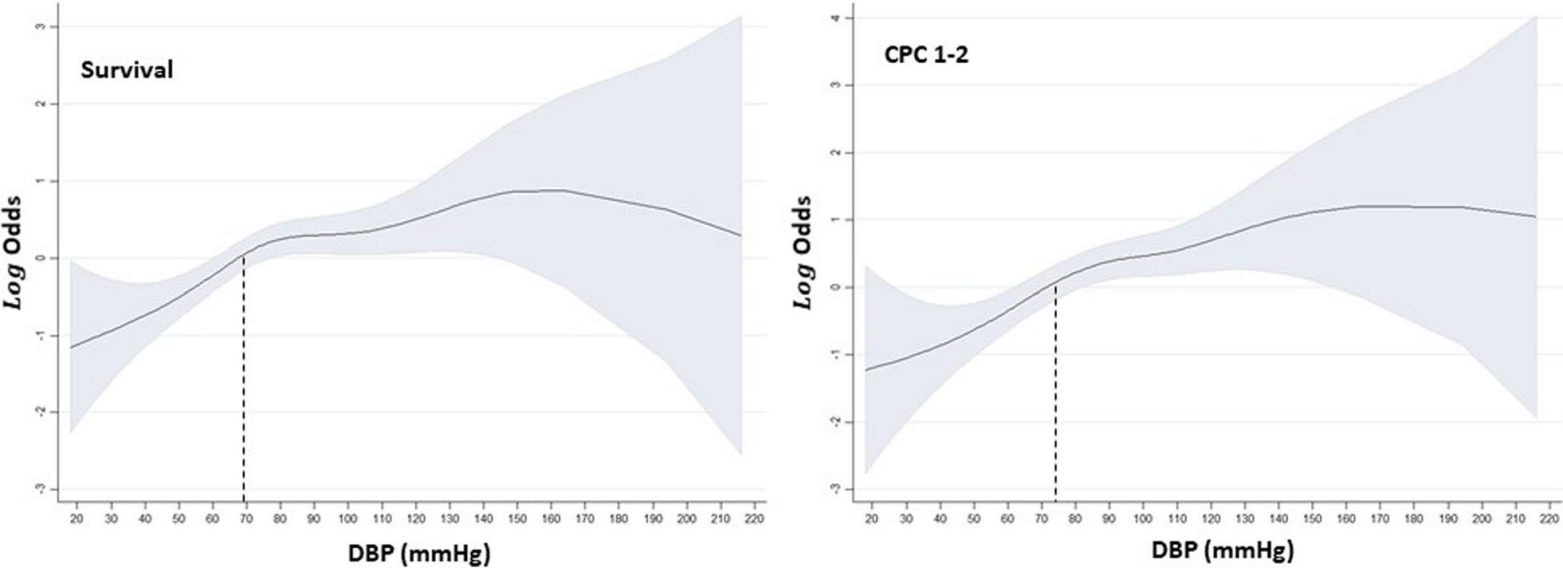
General additive model (GAM) of diastolic blood pressure (DBP) values over outcomes. *GAM* general additive model, *CPC* cerebral performance scale, *DBP* diastolic blood pressure

The relationship between DBP values and favorable outcomes determined by GAM is shown in Fig. 3. GAM revealed a sigmoid curve in both survival and favorable neurologic outcomes. The cut-off values (log odds > 0; i.e., odds > 1) of DBP were around 70–80 mmHg for both survival and favorable neurologic outcomes.

The DBP threshold for better outcomes was further investigated by subgroup analysis according to incremental DBP cut-off points. The unadjusted and adjusted odds ratios of each DBP threshold, when using patients with DBP values lower than each threshold as a reference, are listed in Table 3. The association between DBP and outcomes decreased with increasing DBP cut-off points. In the multivariate analysis, DBP thresholds of 60, 70, and 80 mmHg were associated with favorable neurologic outcomes. When the DBP threshold reached 90 mmHg, the association with favorable neurologic outcomes was no longer present.

**Table 3:** Univariate and multivariate analyses for outcomes according to incremental cut-off values of DBP

	Survival		CPC 1–2	
ROSC DBP (mmHg)	Unadjusted OR (95% CI)	Adjusted OR (95% CI)	Unadjusted (95% CI)	Adjusted OR (95% CI)
DBP > 60	2.48 (1.60–3.83)*	1.96 (1.22–3.15)*	2.90 (1.57–5.33)*	2.15 (1.05–4.41)*
DBP > 70	2.20 (1.50–3.26)*	2.16 (1.41–3.31)*	2.36 (1.44–3.88)*	2.42 (1.32–4.46)*
DBP > 80	1.64 (1.12–2.40)*	1.46 (0.95–2.23)	2.26 (1.14–3.61)*	2.04 (1.14–3.66)*
DBP > 90	1.49 (0.98–2.26)	1.32 (0.83–2.10)	2.03 (1.25–3.30)*	1.84 (0.99–3.39)

*CPC* cerebral performance category, *ROSC* return of spontaneous circulation, *DBP* diastolic blood pressure

^*^Indicates *p*-value < 0.05

### Characteristics of patients with different DBP levels

An exploratory analysis of the characteristics of patients with different DBP levels was performed to evaluate the possible pathophysiological link between DBP and outcomes (Table 4). The DBP cut-off value was set at 80 mmHg according to the results of the threshold analysis. The percentages of survival to discharge (35.8% vs 47.8%, *p* = 0.012), favorable neurologic outcomes (14.6% vs 27.8%, *p* = 0.001), male gender (60.1% vs 69.4%, *p* = 0.043), initial shockable rhythm (28.7% vs 37.8%, *p* = 0.045), and cardiac etiologies (43.7% vs 55.6%, *p* = 0.015) were significantly higher in patients with higher DBP. The prevalence of new-onset post-cardiac arrest complications showed no difference between the two groups.

**Table 4:** Outcomes, characteristics, and post-cardiac arrest complications stratified by DBP

	DBP < 80 mmHg *N* = 268	DBP > 80 mmHg *N* = 180	*p*-value
Age (years)			0.386
< 40	20 (7.5%)	15 (8.3%)	
40–60	67 (25.0%)	55 (30.6%)	
60–80	123 (45.9%)	81 (45.0%)	
> 80	58 (21.6%)	29 (16.1%)	
Origin			0.147
OHCA	215 (80.2%)	154 (85.6%)	
IHCA	53 (19.8%)	26 (14.4%)	
Gender, male	161 (60.1%)	125 (69.4%)	0.043*
DM	109 (40.7%)	74 (41.1%)	0.926
HTN	148 (55.2%)	99 (55.0%)	0.963
CAD	71 (26.5%)	50 (27.8%)	0.764
HF	51 (19.0%)	40 (22.2%)	0.410
COPD	33 (12.3%)	19 (10.6%)	0.560
ESRD	38 (14.2%)	20 (11.1%)	0.343
Malignancy	35 (13.1%)	24 (13.3%)	0.933
Witness	206 (76.9%)	146 (81.1%)	0.283
Bystander CPR	166 (61.9%)	127 (70.6%)	0.060
Initial shockable rhythm	77 (28.7%)	68 (37.8%)	0.045*
Epinephrine dosage (mg)			0.106
0–2	128 (47.8%)	107 (59.4%)	
2–4	70 (26.1%)	39 (21.7%)	
4–6	26 (9.7%)	13 (7.2%)	
> 6	44 (16.4%)	21 (11.7%)	
CPR duration (min)			0.101
< 10	65 (23.1%)	38 (21.1%)	
10–20	75 (28.0%)	52 (28.9%)	
20–30	51 (19.0%)	50 (27.8%)	
> 30	80 (29.9%)	40 (22.2%)	
Cardiac etiology	117 (43.7%)	100 (55.6%)	0.015*
PCI	66 (24.6%)	57 (31.7%)	0.102
Bleeding	69 (25.7%)	46 (25.6%)	0.964
Arrhythmia	121 (45.1%)	66 (36.7%)	0.074
Infection	120 (44.8%)	84 (46.7%)	0.694
Seizure	80 (29.9%)	47 (26.1%)	0.389
Hypokalemia	166 (61.9%)	115 (63.9%)	0.676
Hypoglycemia	26 (9.7%)	22 (12.2%)	0.398
Survival	96 (35.8%)	86 (47.8%)	0.012*
CPC 1–2	39 (14.6%)	50 (27.8%)	0.001*

*DBP* diastolic blood pressure, *OHCA* out-of-hospital cardiac arrest, *IHCA* in-hospital cardiac arrest, *DM* diabetes mellitus, *HTN* hypertension, *CAD* coronary artery disease, *HF* heart failure, *COPD* chronic obstructive pulmonary disease, *ESRD* end-stage renal disease, *CPR* cardiopulmonary resuscitation, *ROSC* return of spontaneous circulation, *PCI* percutaneous coronary intervention, *CPC* cerebral performance category

^*^Indicates *p*-value < 0.05

## Discussion

In this study, we found that the DBP distributions of patients with and without favorable outcomes when discharged differed significantly, while SBP distributions did not. We further proved that DBP was the only independent hemodynamic predictor by stepwise multivariate logistic regression. The correlation between DBP and favorable outcomes was visualized by GAM, which presented a sigmoid curve in both outcomes. GAM and incremental threshold analysis demonstrated that the DBP cut-off value for favorable neurologic outcomes was around 80 mmHg. We further found that the patient subgroup with higher DBP levels had a higher chance of cardiogenic cardiac arrest and initial shockable rhythm.

Current studies regarding the optimal hemodynamic level during post-cardiac arrest care are inconclusive [28]. Several randomized trials also demonstrated no long-term benefit in targeting a higher hemodynamic level [18–20]. However, all these studies focused on SBP and MBP in the post-cardiac arrest period. Only one study found that the lowest DBP during the first 6 h after ICU admission related to the outcomes [22]. Our study was different in that it targeted the hemodynamic level immediately after ROSC, while other studies mainly focused on the ICU period. The benefit of our study design was that it eliminated the confounding effect of inotropic agents and TTM, which strongly affect hemodynamic levels due to the inconsistent use of inotropic agents and different TTM protocols. Other studies similar to our study reported a relationship between outcomes and early hemodynamic level after resuscitation [1, 3, 13]. However, these studies were conducted on patients with pre-hospital ROSC, and only SBP was addressed.

DBP and SBP have different physiologic features. SBP is more sensitive to large artery compliance, cardiac contractility, and intravascular volume change than DBP [29–31]. An initial target SBP of 90 mmHg is commonly used in circulatory shock. However, DBP is more related to peripheral vascular resistance if aortic valve function is intact [29, 31–33]. Due to decreasing peripheral vascular tone in sepsis, some studies suggested DBP as a more reliable predictor of better outcomes of septic shock patients than SBP [32, 33]. DBP is also strongly related to coronary perfusion during the diastolic phase [34]. Better coronary perfusion is a key determinant of successful resuscitation [35]. Greater DBP values during CPR were associated with a greater chance of ROSC in children and a porcine model [23–25].

Our study found that higher DBP values in the early post-resuscitation stage correlated with good outcomes. Two theories might explain the underlying mechanism of the correlation between DBP and the favorable prognosis of cardiac arrest patients. One theory assumes that DBP is correlated with coronal perfusion pressure. Therefore, a better DBP directly leads to better myocardial perfusion and a greater chance of survival. The other theory assumes that the DBP level represents the severity of post-cardiac arrest syndrome [31, 36]. Post-cardiac arrest syndrome, including systemic ischemia and reperfusion injury, is similar to septic shock and can cause peripheral vascular tone loss [36]. As mentioned above, DBP can represent peripheral vascular resistance as well as post-cardiac arrest syndrome. Cardiac arrest patients with initial shockable rhythm and cardiac etiologies were assumed to have shorter no-flow time, less ischemic stress, and less post-cardiac arrest syndrome than those with non-shockable and non-cardiac etiologies [37–39]. This hypothesis correlated with our study finding that patients with a higher DBP level had a greater chance of having initial shockable rhythm and cardiogenic cardiac arrest (Table 4). Males had a higher risk of cardiogenic arrest, as seen by the higher correlation between male gender and DBP value in this study. Incidences of other new-onset complications, including bleeding, arrhythmia, sepsis, and seizure, were not different. These severe complications might have multiple causes and cannot be simply explained by the DBP level alone.

The study findings have some clinical implications. First, SBP and MBP are the primary hemodynamic targets for circulatory shock and organ perfusion. However, these two parameters physiologically represent only part of the systemic hemodynamics. Our study showed that ROSC DBP is a more reliable hemodynamic parameter than SBP and can reflect the level of post-cardiac arrest syndrome. DBP can also serve as a surrogate marker of systemic vascular resistance if advanced hemodynamic parameters, such as pulmonary artery catheters or pulse contour cardiac output catheters, are not available. Second, our study suggested that a DBP around 80 mmHg might be a possible clinical target for better outcomes. Theoretically, a higher target BP might improve cerebral blood flow according to the right shift of cerebral autoregulation after cardiac arrest, but recent randomized trials showed no difference in long-term outcomes [18–20]. However, the hemodynamic target of these studies was MBP. Our study pointed out a possible DBP target in post-resuscitation care. Future studies emphasizing the optimal DBP level during the post-resuscitation period should be conducted.

This study has several limitations. First, this was a retrospective study, and the study population was retrieved from nine medical centers. Differences among facilities in TTM protocols or post-cardiac arrest management, including nutritional support, and glucose management, could not be addressed in this study. Second, several important prognosis factors, including lactate level, blood gas analysis, urine output, and echocardiography results, were not collected due to the limited registry design. In this study, we aimed to predict patients’ outcomes based on variables at the time of ROSC. Therefore, several post-cardiac arrest care factors, TTM factors and TTM complications were not included in the prediction model. Third, we only evaluated the hemodynamic level at one time point in this study. The complexity of the relationship between hemodynamics, outcomes, inotropic agents, and TTM in the later post-resuscitation period was not addressed. Fourth, we sought to define an optimal hemodynamic level for better outcomes. However, we could only prove the relationship between DBP values and favorable outcomes through statistical analysis. It is unknown whether higher DBP levels lead to better outcomes or patients with better outcomes have higher DBP levels. A further prospective study is needed to estimate the optimal DBP level for post-cardiac arrest patients. Finally, data on the patients’ long-term outcomes after discharge were not available from the registry.

## Conclusion

ROSC DBP is an independent hemodynamic predictor of better outcomes. A higher DBP value correlated with a higher prevalence of initial shockable rhythm and cardiogenic cardiac arrest. This finding verifies the hypothesis that DBP level can represent the severity of ischemic stress or post-cardiac arrest syndrome. A further prospective study is needed to determine the optimal DBP value of post-cardiac arrest patients.

## Data Availability

The data that support the findings of this study are available from the *Taiwan Society of Emergency and Critical Care Medicine,* but restrictions apply to the availability of these data, which were used under license for the current study, and so are not publicly available. Data are, however, available from the authors upon reasonable request and with permission of the *Taiwan Society of Emergency and Critical Care Medicine*.

## Abbreviations

MBP: Mean blood pressure
SBP: Systolic blood pressure
DBP: Diastolic blood pressure
ICU: Intensive care unit
ROSC: Return of spontaneous circulation
TTM: Targeted temperature management
OHCA: Out-of-hospital cardiac arrest
IHCA: In-hospital cardiac arrest
NIBP: Non-invasive blood pressure
ED: Emergency department
PCI: Percutaneous coronary intervention
ECMO: Extracorporeal membrane oxygenation
CPC: Cerebral performance category
ROC: Receiver operating characteristic
GAM: Generalized additive model
DM: Diabetes mellitus
HTN: Hypertension
CAD: Coronary artery disease
HF: Heart failure
COPD: Chronic obstructive pulmonary disease
ESRD: End-stage renal disease
CPR: Cardiopulmonary resuscitation
PEA: Pulseless electrical activity
VT: Ventricular arrhythmia
VF: Ventricular fibrillation
